# Genetics of depressive symptoms in adolescence

**DOI:** 10.1186/s12888-017-1484-y

**Published:** 2017-08-31

**Authors:** Hannah Sallis, Jonathan Evans, Robyn Wootton, Eva Krapohl, Albertine J Oldehinkel, George Davey Smith, Lavinia Paternoster

**Affiliations:** 1 MRC Integrative Epidemiology Unit, Population Health Sciences, Bristol Medical School, University of Bristol, Barley House, Oakfield Grove, Bristol, BS8 2BN UK; 2 Centre for Academic Mental Health, Population Health Sciences, Bristol Medical School, University of Bristol, Bristol, UK; 3 School of Experimental Psychology, University of Bristol, Bristol, UK; 4 MRC Social, Genetic and Developmental Psychiatry Centre, Institute of Psychiatry, Psychology and Neuroscience, King’s College London, London, UK; 5 Interdisciplinary Center Psychopathology and Emotion Regulation, University of Groningen, University Medical Center Groningen, Groningen, The Netherlands

**Keywords:** ALSPAC, Adolescent depression, Heritability, GWAS

## Abstract

**Background:**

Despite many attempts to understand the genetic architecture of depression, little progress has been made. The majority of these studies, however, have been carried out in adults and do not account for the potential influence of development.

**Methods:**

The Avon Longitudinal Study of Parents and Children (ALSPAC) is a longitudinal pregnancy cohort which recruited participants between April 1991 and December 1992. Analyses were replicated in two independent European cohorts. Genome-wide complex trait analysis (GCTA) software was used to investigate SNP-heritability (h^2^_SNP_) of depression across adolescence, the role of puberty was investigated by stratifying these estimates according to pubertal onset. Genome-wide association studies were performed to identify genetic variants associated with depression at different stages of development.

**Results:**

Heritability was estimated between the ages of 11 and 18 with sample sizes ranging from 3289 to 5480. Heritability was low with an apparent peak was found at age 13 (h^2^ = 0.17, *p* = 0.006). Confidence intervals around these estimates suggest an upper-bound to h^2^_SNP_ of around 30%. A variant located on chromosome 7 was found to be associated with depressive symptoms at age 13 in ALSPAC (rs138191010: β = 0.142, *p* = 2.51 × 10^−8^), although this was not replicated.

**Conclusions:**

Although power is a potential limitation, the observed patterns provide interesting hypotheses surrounding the heritability of depression at different developmental stages. We found substantially lower estimates for depressive symptoms at age 11 (0.07) compared to those previously estimated in adults (0.21). We also found a peak in heritability at age 13. These findings suggest environmental factors are likely to be more important in the aetiology of depressive symptoms in early adolescence than in adulthood.

**Electronic supplementary material:**

The online version of this article (10.1186/s12888-017-1484-y) contains supplementary material, which is available to authorized users.

## Background

Despite many attempts to understand the genetic architecture of depression, progress has been slow, particularly when compared with other complex disorders. The Psychiatric Genomics Consortium (PGC) recently identified 108 risk loci for schizophrenia [1], whereas corresponding analyses performed for major depressive disorder (MDD) failed to find any robustly associated loci [2]. More recently, however, studies have utilised data from the UK Biobank and 23andMe which resulted in discovery samples of over 100,000 participants of European ancestry [3, 4]. The China, Oxford and Virginia Commonwealth University Experimental Research on Genetic Epidemiology (CONVERGE) consortium applied sparse whole genome sequencing to a sample of over 5000 cases of major depression and 5000 controls amongst Han Chinese women [5]. These studies report finding several genetic variants associated with depressive symptoms measured in adults.

Both phenotypic and/or genetic heterogeneity may contribute to the slow progress in identifying genetic loci robustly associated with depression [6]. Work has focused on dissecting the phenotype, with studies focusing on age at onset or subgroups including early onset or recurrent depression, in an attempt to understand the genetic architecture [6–8].

Heritability is the proportion of variation in a phenotype attributable to genetic differences. A meta-analysis of twin studies estimated the heritability of MDD in adults to be 0.37 (95% CI: 0.31, 0.42) [9], while recent work by the PGC used SNP-based methods implemented by the Genome-wide Complex Trait Analysis (GCTA) software [10] to estimate a common SNP-heritability (h^2^_SNP_) of 0.21 [11]. Although these estimates may at first appear inconsistent, the estimate from twin studies is expected to be higher as they take into account variation across the entire genome, whereas h^2^_SNP_ estimates incorporate only common variants captured by SNPs included on genotyping platforms.

The majority of these studies of depression have been carried out in adults. However, a recent publication by Nivard et al. [12] investigated symptoms of anxiety and depression across the lifecourse using data from the Netherlands Twin Registry (NTR), suggesting heritability might vary according to age. The study combined data from 49,524 twins whose ages ranged from 3 to 63. Heritability estimates calculated in this study showed fluctuations from early life until the early 20s (highest at 0.69, aged 3), after age 20 heritability remained relatively stable at around 0.5.

Nivard et al. [12] found a substantial genetic component to childhood depression, with a distinct drop in heritability from 0.58 to 0.37 at age 12. Estimates increased again throughout adolescence and reached a peak of 0.53 at age 18. This is an interesting contrast to findings from many other twin studies which suggest a minor genetic component to depression in early life and increasing heritability with age, the heritability in late adolescence being comparable with that of adult depression [13–17]*.* However, the drop in heritability found in the Nivard study might be confounded by a switch in reporting method, as depression measures switched from parent- to self-reported questionnaires.

In addition to coinciding with a switch in reporting method, the timing of this change in heritability is interesting as it coincides with pubertal onset. At age 12, many participants are likely to be nearing the onset of puberty, if they have not entered it already. Pubertal status and depression are highly associated, with a 1 year prevalence of depression of around 1% in pre-pubertal children, rising to 4–5% in mid to late adolescence [18]. Prior to puberty the prevalence of depression is similar in both males and females, however after pubertal onset this rises more rapidly among females. This has prompted some to hypothesise that changes during puberty, or the timing of pubertal onset, could be related to the onset of depressive symptoms [19–21]. Although mechanisms are unclear, biological theories suggest earlier puberty and the hormonal changes associated with this, such as increasing levels of dehydroepiandrosterone (DHEA), may act as a trigger for some underlying vulnerability [22, 23]. Alternatively, psychosocial theories hypothesise that depression is a social consequence of the early onset of puberty compared to peers [24].

We aim to investigate patterns of heritability across adolescence using data from the Avon Longitudinal Study of Parents and Children (ALSPAC), which has both parent- and self-reported measures of depressive symptoms available at regular intervals during this important developmental period.

ALSPAC is a prospective birth cohort started in the early 1990s, which holds information on a number of measures, including depression and pubertal status. This includes a combination of parent- and self-reported depression measures at a variety of time points, which enabled us to investigate the change in heritability found by Nivard et al. [12]. Genotyping data are available on a large number of ALSPAC participants, enabling us to estimate h^2^_SNP_ directly at different ages using restricted maximum likelihood (REML) methods and to attempt to identify genetic variants associated with depression measures across a range of ages.

## Methods

### Avon longitudinal study of parents and children (ALSPAC)

ALSPAC is a longitudinal pregnancy cohort which aimed to recruit all pregnant women in the former county of Avon with an expected due date between April 1991 and December 1992. Detailed information has continued to be collected on mothers, partners and children in the cohort, this process has been described in detail elsewhere [25, 26]. Ethical approval for the study was obtained from the ALSPAC Ethics and Law Committee and the Local Research Ethics Committees. A fully searchable data dictionary with information on all available measures is available at http://www.bris.ac.uk/alspac/researchers/data-access/data-dictionary/.

### Depression measures

Symptoms of depression in the ALSPAC children were measured throughout childhood and adolescence using a combination of both parent- and self-reported responses to the Short Moods and Feelings Questionnaire (SMFQ) [27]. The SMFQ consists of 13 phrases relating to the feelings and actions of participants in the previous 2 weeks, which they are asked to rate as ‘most of the time’, ‘some of the time’ or ‘not at all’ (scoring 2,1 or 0, respectively). A higher score on the SMFQ corresponds to a greater level of depressive symptoms.

Timing of SMFQ measurements is described in detail in Additional file 1: Materials and Methods. For the purposes of these analyses and to avoid bias from shift in reporting, we focus on self-reported responses.

### Pubertal status

Pubertal status was regularly measured in ALSPAC, between 7.5 to 18 years. Tanner Stages were used to measure pubertal stage in both males and females [28, 29]. In this analysis we have used the Tanner Stage corresponding to pubic hair development for both males and females to assess pubertal onset.

Further details on the derivation of pubertal onset are described in Additional file 1: Materials and Methods.

### Genotyping and GRM

Directly genotyped data were available on 8237 children and 477,482 SNPs. A genetic relatedness matrix (GRM) was estimated for this sample. Full details can be found in Additional file 1: Materials and Methods.

### Statistical analysis

#### Calculating h^2^_SNP_

*h* ^*2*^_*SNP*_ was calculated using the REML method implemented within the GCTA software [10]. Estimates of heritability were calculated and plotted for each age at which we had a self-reported measure of depressive symptoms. Given our interest in investigating whether there is a drop in heritability around puberty as seen in Nivard et al. [12], we focused on ages 11, 13, and 18. In addition to estimating h^2^_SNP_ at each of these ages, we performed exploratory analyses stratified by sex and also pubertal status when participants were aged 11 and 13.

For each of our h^2^_SNP_ estimates, analyses were adjusted for age. Where analyses were performed on the overall sample, we also adjusted for sex.

As a sensitivity analysis, we re-calculated h^2^_SNP_ estimates for our main ages of interest, restricting to participants with data on pubertal status. Given that the distribution of SMFQ scores varied according to age, we also applied a quantile normal transformation and used these transformed scores in the analysis.

Power calculations were performed for our analyses using the GCTA-GREML power calculator available at http://cnsgenomics.com/shiny/gctaPower/ [30].

#### Genome-wide association analysis (GWAS)

Genome-wide association analyses were carried out for self-reported depressive symptoms at ages 11, 13, and 18 and performed using SNPTEST v2.5 [31]. Analyses were adjusted for age and sex. Both the REML and GWAS analyses were restricted to participants of a European ancestry.

#### Replication

A look up of any SNP achieving genome-wide significance (*p* < 5 × 10^−8^) in this analysis was performed in both the TRacking Adolescents’ Individual Lives Survey (TRAILS) [32, 33] and the Twins Early Development Study (TEDS) [34]. Information on both cohorts can be found in Additional file 1: Materials and Methods.

## Results

The number of ALSPAC participants with complete information on both depression and genotype ranged from 5480 at age 11 to 3289 at age 18 (those also with puberty data were lower, 3759 and 1724 at 11 and 18, respectively). There were slightly more females than males and this increased slightly with age, (49.4% males at age 11 reducing to 43.0% at 18, Table 1).

**Table 1:** Sample Characteristics

		Age 11 (*N* = 5480)	Age 13 (*N* = 5055)	Age 18 (*N* = 3289)
Mean age in months (SD)		127.7 (3.02)	153.6 (2.73)	213.9 (4.43)
Males N (%)		2706 (49.4)	2462 (48.7)	1414 (43.0)
SMFQ score (LQ-UQ)^1^		3 (1–6)	3 (1–5)	5 (3–9)
Subset with puberty data - N		3759	3147	1724
Puberty – number post-onset (%)	Overall	1402 (37.3)	2913 (92.6)	1723 (99.9)
	Male	525 (31.9)	1240 (88.5)	675 (100.0)
	Female	877 (41.5)	1673 (95.8)	1048 (99.9)

^1^ Median child reported SMFQ score with lower and upper quartiles reported due to skew in score

Proportion of participants in the pre-pubertal category differed according to sex. At ages 10 and 13, there were a greater proportion of females who had experienced the onset of puberty compared with males (*p* < 0.001 in both cases, Table 1). The proportion of the overall sample reaching onset increased from 37.3% at age 10 to 92.6% at age 13. By the time the sample was aged 18, 99% of participants reported onset of puberty.

### Estimating h^2^_SNP_

#### Overall sample

**Fig. 1:**
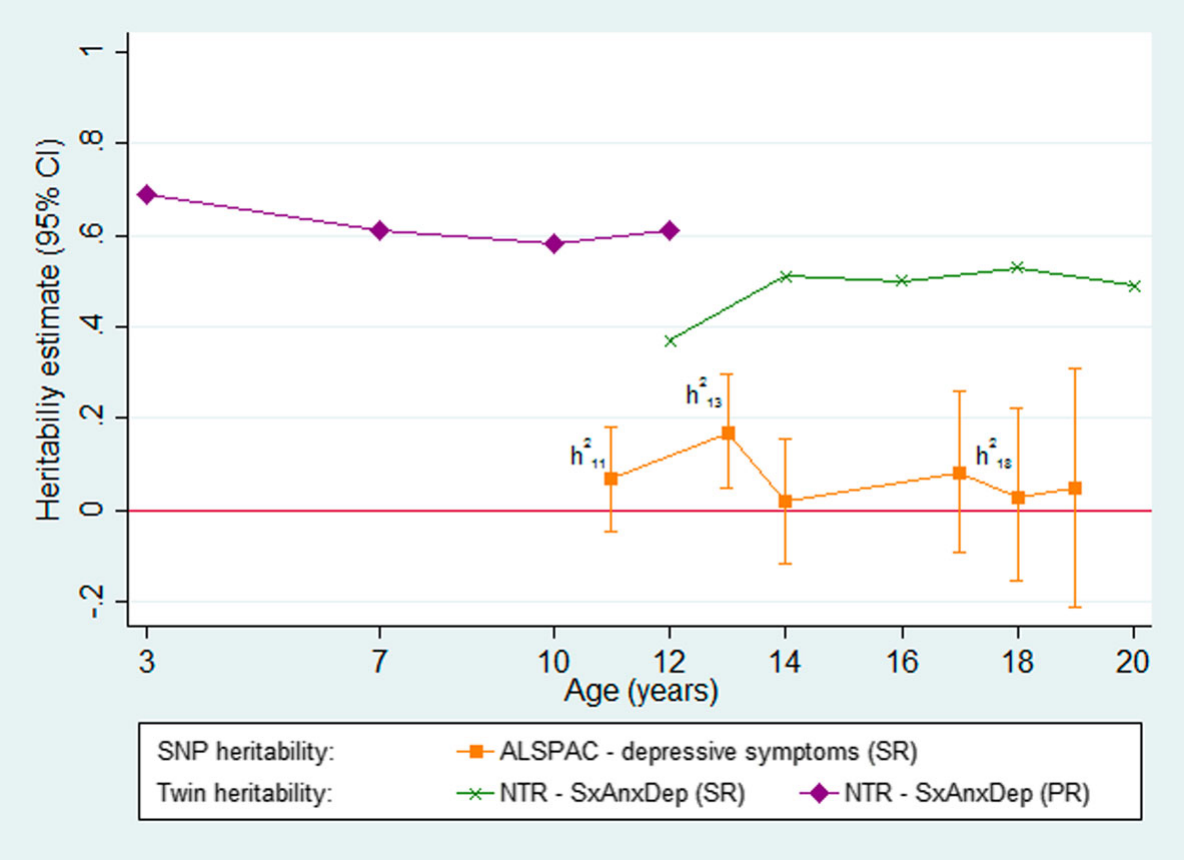
Heritability estimates of depressive symptoms measured during childhood and adolescence in NTR and ALSPAC

h^2^_SNP_ estimates of child self-reported SMFQ ranged from 2% to 17%, with a peak around age 13 (h^2^ = 0.17, *p* = 0.006) (Fig. 1, Table 2). However standard errors for these estimates are large and overlapping, with only the estimate at age 13 showing robust evidence for a non-zero h^2^_SNP_. The pattern is consistent across both parent- and self-reported measures, although parent-reported measures suggested lower estimates of h^2^_SNP_ at comparable time points (Figs 1 and Additional file 1: Fig. S1). Given our interest in investigating the pattern of effects found by Nivard et al. [12], we focus on self-report data from 3 time points – ages 11, 13 and 18.

**Table 2:** Heritability estimates of child-reported SMFQ scores at the main ages of interest^1^

		Age 11			Age 13			Age 18		
		N	h^2^ (se)	*P*-value	N	h^2^ (se)	*P*-value	N	h^2^ (se)	*P*-value
Overall sample		5480	0.07 (0.06)	0.244	5055	0.17 (0.06)	0.006	3289	0.03 (0.10)	0.712
Male		2706	0.04 (0.12)	0.760	2462	0.30 (0.13)	0.020	1414	<0.001 (0.22)	1.000
Female		2774	0.07 (0.12)	0.570	2593	0.03 (0.12)	0.790	1875	0.18 (0.17)	0.286
Stratified by puberty										
Male	Pre	1123	<0.001 (0.28)	1.000	161	0.05 (1.96)	0.980	-	-	
	Post	525	0.81 (0.59)	0.156	1240	0.43 (0.27)	0.108	-	-	
Female	Pre	1234	0.39 (0.26)	0.134	73	1.00 (4.28)	0.784	-	-	
	Post	877	0.11 (0.37)	0.758	1673	0.10 (0.19)	0.592	-	-	

^1^ All analysis adjusted for age (in months), overall sample additionally adjusted for gender

#### Stratified by sex

When we perform exploratory sex-stratified analysis, the h^2^_SNP_ peak occurs at age 13 in males (h^2^ = 0.30, *p* = 0.020), but at 18 in females (h^2^ = 0.18, *p* = 0.286), whereas the h^2^_SNP_ estimates for males at age 18 and females at 13 are close to zero (Table 2). However, standard errors for these estimates are large and overlapping. If real, these differences might be explained by differences in pubertal timing of males and females, we therefore investigated the effect of puberty on h^2^_SNP_ estimates.

#### Stratified by puberty

To investigate whether pubertal timing could explain the differences in h^2^_SNP_ estimates at different times in males and females we performed exploratory analysis stratified according to pre- and post-onset of puberty.

When we stratify the results by pubertal stage, at age 11 we see increased heritability among males post-onset (h^2^ = 0.81, *p* = 0.156) and females pre-onset (h^2^ = 0.39, *p* = 0.134); a similar pattern is seen at age 13 (Table 2). However, the sample sizes in these stratified analyses are small, resulting in large standard errors and thus results should be interpreted with caution.

We investigated whether timing of puberty had an effect on h^2^_SNP_ by adjusting the female estimates for age at both assessment and menarche. The pattern of estimates remained relatively consistent, with the majority of estimates becoming larger after controlling for age at menarche (Additional file 1: Table S1).

### Sensitivity analysis

#### Complete case analysis

When we re-estimated overall h^2^_SNP_ at each of our ages of interest using only participants with complete data (i.e. pubertal stage), the estimates for ages 11 and 13 became smaller (<0.1% and 4.1% respectively), while h^2^_SNP_ at age 18 remained of a similar level (3.3%). However, the number of participants in the younger age groups was substantially reduced, those with data available at age 18 remained similar.

#### Transformed data

Given that the SMFQ data were somewhat skewed, and the skew appeared to increase with age (Additional file 1: Fig. S2), we applied a quantile normalisation transformation at each age. The pattern of heritability estimates remained relatively constant. Of our 3 main ages of interest, we saw a slight increase in estimated heritability at age 11 and 18 (0.08 and 0.1 respectively), the peak seen at 13 remained (h^2^ = 0.15) (Additional file 1: Table S2).

#### Genome-wide association studies

**Fig. 2:**
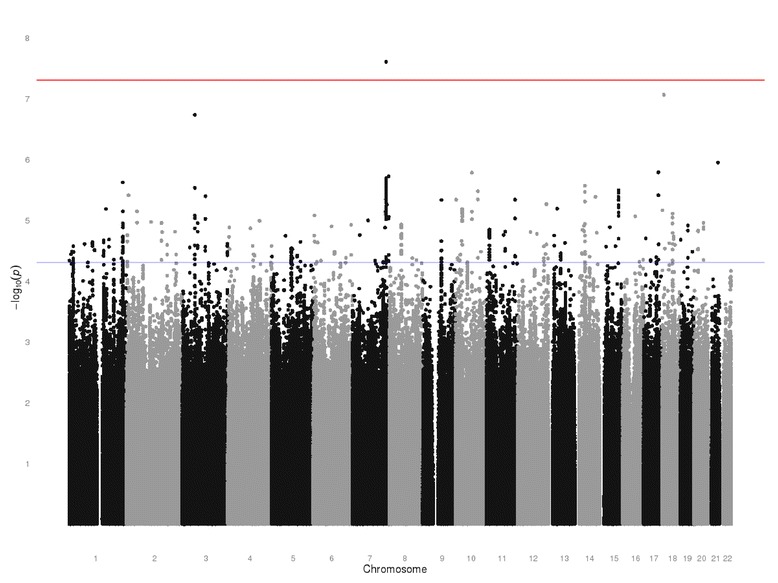
Manhattan plot showing associations from GWAS of SMFQ at 13 years

A genome-wide association study (GWAS) was performed for each of our ages of interest. At age 11 and age 18 we found no variants achieving genome-wide significance levels (*p* ≤ 5 × 10^−8^). At age 13, however, we found evidence of an association between a well-imputed (information score = 0.88) variant on chromosome 7 (rs138191010 – risk allele: T, β = 0.142, se = 0.03, *p* = 2.51 × 10^−8^) and depressive symptoms (Fig. 2, Additional file 1: Fig. S3). Sex-stratified analyses found no robust associations, although the direction of effect with rs138191010 was consistent (Females: β = 0.134, se = 0.04, *p* = 1.38 × 10–^4^; Males: β = 0.150, se = 0.04, *p* = 5.71 × 10^−5^). There was also no strong evidence of an association between rs138191010 and depressive symptom scores at either age 11 or 18, however the direction of effect was consistent across all time points (Age 11: β = 0.065, se = 0.03, *p* = 0.009; Age 18: β = 0.062, se = 0.03, *p* = 0.054).

A sensitivity analysis using quantile normalised scores at age 13 found strong evidence of an association (β = 0.135, se = 0.03, *p* = 6.7 × 10^−8^).

A look up of this variant in both the TRAILS and TEDS cohorts failed to replicate the finding (Additional file 1: Fig. S4).

## Discussion

This study aimed to build on work by Nivard et al. [12] by attempting to unpick the heritability of depression across adolescence and investigating the role of puberty. Unlike the Nivard study, which found that heritability decreased substantially at age 12, our estimates, although lower, appeared to peak close to this time at age 13 (h^2^_twin_: 0.37 vs h^2^_SNP_:0.17). Although Nivard et al. [12] offered several explanations for this, including puberty, they noted that it coincided with a switch in questionnaires from parent to self-report, acknowledging that the decrease may be an artefact of the change in reporting method.

Nivard et al. [12] found increased heritability estimates when using parent-reported depressive symptoms, however, in ALSPAC these estimates were consistently lower than for self-reported symptoms. Regardless of rater, and in both raw and transformed data, we found a peak in heritability around age 13. With raters only overlapping at one time point, it seems likely that the drop in heritability seen in the Nivard paper is related to change in reporting method.

Within ALSPAC both parent and self-reported depressive symptoms are available, enabling us to remove this source of confounding from estimates, however, confounding according to timing of puberty remains. We investigated this by stratifying on pubertal status, enabling us to explore patterns of heritability according to age and pubertal status. However, our sample size was reduced somewhat when running these exploratory analyses and restricting to those with data on puberty, and no firm conclusions could be made.

We dichotomised pubertal stage into pre- and post-pubertal onset according to Tanner stage. At age 11 around 30% of males had reached puberty, compared to approximately 40% of females. At this age, heritability estimates were highest among males who had reached puberty (h^2^ = 0.81, *p* = 0.156) and pre-pubertal females (h^2^ = 0.39, *p* = 0.134). Given that males generally experience puberty later than females, it is possible that these differences in heritability are related to differences in timing of puberty and changes in environmental rather than genetic risk. One explanation would be that the social consequences of earlier puberty are opposite for males and females, with earlier puberty being protective in boys reducing environmental risk and thereby increasing the relative contribution of genetic risk. While earlier puberty could increase environmental risk in females, hence reducing the relative contribution of genetic risk at this age.

When stratifying analyses on sex, regardless of pubertal status, heritability was increased for males at age 13 (h^2^ = 0.30) and females at 18 (h^2^ = 0.18). This increased male estimate appears to be driving the overall peak in heritability (h^2^ = 0.17) at 13, although a sex-stratified GWAS found no SNPs achieving genome-wide significance.

We had good power when using the entire sample (power at age 11:84%; 13:78%; 18:42%) to detect a true heritability of 17%. Despite little statistical evidence for non-zero heritability at most ages, the confidence intervals estimated for age 11 exclude 21%, suggesting that our results demonstrate h^2^_SNP_ at this age is lower than that previously reported for adult depression. Though this could be explained by phenotype definition, rather than biological differences per se, as we used self-reported measures of depressive symptoms rather than detailed diagnostic interviews. When performing stratified analyses, power will be diminished further, however, the pattern of results observed generate interesting hypotheses that could be followed up in larger datasets.

While Nivard et al. [12] found high heritability estimates among their younger age groups, estimates at age 11 in our study do not overlap with these. However, it is important to remember SNP-based and twin heritability are different. We would expect twin estimates to be higher simply due to the study design, which relies on closely related individuals. Some of this inflation in h^2^ is real, due to shared rare genetic variants, whereas some is due to shared common environment, and therefore false inflation of the estimates. Our analyses are restricted to unrelated individuals in an attempt to prevent inflation of our h^2^_SNP_ estimates due to shared environment. Additionally, our analyses incorporate only variants captured on the genotyping platform used, and is an estimate of the heritability we could potentially estimate through GWAS.

Although we expect to see higher estimates from Nivard et al. [12] in general, we can compare the patterns of heritability across time in the two studies. Nivard et al. [12] report a decline in heritability during childhood and adolescence, however the results shown in ALSPAC do not support this. Our results are relatively stable from age 11–18, with the exception of a peak at around age 13.

It is also important to consider selective drop out when thinking about these patterns of heritability. Those with increased depressive symptoms may be more likely to drop out, leading to declines in heritability. However, no robust evidence of association between a genetic risk score for depression and dropout was found in our sample (*p* = 0.67).

A GWAS in ALSPAC found evidence of association between a variant on chromosome 7 (rs138191010) and depressive symptoms at age 13 (when we also see greatest heritability). However, this was not replicated in TRAILS (which found weak evidence of an association in the opposite direction) or TEDS (which found no robust evidence of an association). Both ALSPAC and TEDS used the SMFQ to assess depression, while the Youth Self Report was used in TRAILS. When restricting to just ALSPAC and TEDS, which used a consistent measure of depression, there remained no strong evidence of an association. It is therefore possible that this is a false positive.

### Limitations

Although our analyses, in particular those stratifying on pubertal status, may lack power, the patterns observed provide interesting hypotheses surrounding the heritability of depression at different developmental stages, and provide pilot data that can be utilised to design well-powered studies to investigate these hypotheses.

## Conclusion

We estimated heritability of depression at several points through late childhood and across adolescence and found an apparent peak in heritability at age 13, confidence intervals around the heritability estimates throughout these ages suggest an upper-bound to h^2^_SNP_ of around 30%. Our results indicate that sex and pubertal onset are likely to affect heritability estimates. We also found differences between previous h^2^_SNP_ estimates calculated in adults (0.21) and our lower estimates for depressive symptoms found at age 11 (0.07). A variant located on chromosome 7 was found to be associated with depressive symptoms at age 13 in ALSPAC, although this was not replicated in other European studies.

## Additional file

Additional file 1: This file contains Additional materials and methods, Tables S1-S2 and Figs. S1-S3. (DOCX 0.99 mb)

## Abbreviations

ALSPAC: Avon Longitudinal Study of Parents and Children
DHEA: Dehydroepiandrosterone
GCTA: Genome-wide Complex Trait Analysis
GRM: Genetic Relatedness Matrix
GWAS: Genome-Wide Association Study
h^2^_SNP_: SNP-heritability
MDD: Major Depressive Disorder
NTR: Netherlands Twins Registry
PGC: Psychiatric Genomics Consortium
REML: Restricted Maximum Likelihood
se: Standard Error
SMFQ: Short Moods and Feelings Questionnaire
TEDS: Twins Early Development Study
TRAILS: TRacking Adolescents Individual Lives Study
