# Supplementary material for: Genetics of depressive symptoms in adolescence
Source: BMC Psychiatry. 2017 Aug 31;17:321. doi: 10.1186/s12888-017-1484-y (PMC5580280; doi:10.1186/s12888-017-1484-y)
Supplement: Additional file 1: — This file contains Additional materials and methods, Tables S1-S2 and Figs. S1-S3. (DOCX 0.99 mb) [file 12888_2017_1484_MOESM1_ESM.docx]

**Additional file 1**

**Genetics of depressive symptoms in adolescence**

Hannah Sallis PhD^1,2,3^, Jonathan Evans MD^2^, Robyn Wootton MSc^3^, Eva Krapohl MSc^4^, Albertine J Oldehinkel PhD^5^, George Davey Smith DSc^1^, Lavinia Paternoster PhD^1^

1. MRC Integrative Epidemiology Unit, Population Health Sciences, Bristol Medical School, University of Bristol, UK
2. Centre for Academic Mental Health, Population Health Sciences, Bristol Medical School, University of Bristol, Bristol, UK
3. School of Experimental Psychology, University of Bristol, Bristol, UK
4. MRC Social, Genetic and Developmental Psychiatry Centre, Institute of Psychiatry, Psychology and Neuroscience, King's College London, London, UK
5. Interdisciplinary Center Psychopathology and Emotion Regulation, University of Groningen, University Medical Center Groningen, The Netherlands

**Additional file 1 includes:**

Additional Materials and Methods

References

Additional Tables S1-S2

Additional Figures S1-S4

**Additional Materials and Methods**

Measures

SMFQ

Self-reported responses to the SMFQ were collected at 6 time points during adolescence, at 10.5, 12.5, 13.5, 16.5, 18, and 18.5 years of age. For the majority of analyses we focus on three time points (10.5, 12.5, and 18 years), which, for clarity of reporting, we refer to as ages 11, 13 and 18. Parent-reported responses to the SMFQ were available at 4 time points, collected when participants were aged 10, 12, 13, and 17 years, however the majority of these were not measured concurrently with the child report questionnaires.

SMFQ total scores were calculated by summing across each of the 13 items to create a score ranging between 0 and 26 for each participant.

Puberty

Although both scales of the Tanner Scale were measured (relating to pubic hair and genitalia development), a large proportion of males appeared to move backwards in developmental stage according to the genitalia scale so this part of the scale was dropped due to unreliable reporting. Thus in order to be consistent across all participants, we have used only the Tanner Stage corresponding to pubic hair development for both males and females to assess pubertal onset.

The Tanner Stage scale measures pubertal development on a scale of 1 to 5, with 1 being pre-adolescent and prior to the onset of puberty, and 5 being mature adult stage. We dichotomised puberty into pre- and post-onset for use in our analysis. Pubertal status was coded as pre-onset for the ages at which Tanner Stage was recorded as 1, however, once Tanner Stage was reported to be >1 we coded all subsequent age points as post-onset.

In order to make the measure of pubertal status relevant to the corresponding SMFQ, we restricted relevant analyses to participants who did not regress on the Tanner scale at any point in time, and whose depression and puberty measures were collected as close in time as possible and within 12 months of one another.

Genotyping and GRM

ALSPAC children were genotyped using the Illumina HumanHap550 quad chip genotyping platforms by 23andme subcontracting the Wellcome Trust Sanger Institute, Cambridge, UK and the Laboratory Corporation of America, Burlington, NC, US. Following quality control (individual call rate > 0.97, SNP call rate > 0.95, MAF > 0.01, HWE > 1e-7, cryptic relatedness within children IBD < 0.1, non-European clustering individuals removed) 8,237 children were retained with 477,482 SNP genotypes in common between them. SNPs were flipped to forward strand and haplotypes were estimated on the combined sample using ShapeIT (v2.r644). Imputation was performed using Impute V2.2.2 against the 2186 ancestrally diverse reference haplotypes in the Dec 2013 release of the 1000 genomes reference haplotypes (Version 1 Phase 3) (McMahon et al. 2015). This resulted in 28,699,419 imputed SNPs available for analysis. SNPs with MAF<0.01 and imputation info score <0.3 were filtered, leaving 9,092,716 SNPs included in the GWAS.

A genetic relatedness matrix (GRM) was estimated using genotype probabilities for autosomal SNPs imputed against the 1000 Genomes reference panel, confined to SNPs present on the HapMap3 reference panel and with an imputation info score of >0.8. Restricting to these SNPs ensures good genomic coverage whilst reducing bias due to the correlation between the increased numbers of variants present in the 1000 Genomes imputation (Speed et al. 2012; Lee et al. 2013; Speed et al. 2013). A GRM measures the genetic similarity of pairs of unrelated individuals. Close relatives (fourth cousins or closer – relatedness of 0.025 or higher) are removed from this matrix in order to reduce confounding due to shared environment, using unrelated individuals also makes the estimate of genetic variance due to SNPs directly comparable to results from GWAS (Visscher et al. 2014). In addition, removing related individuals minimises issues with causal variants being captured by pedigree information rather than being tagged by genotyped SNPs (Yang et al. 2010). Linkage disequilibrium between genotyped SNPs and unknown causal variants tagged by these enables us to estimate the variance of a phenotype explained by any tagged variant.

Genetic information for replication cohorts

TRAILS is a longitudinal cohort based in The Netherlands investigating adolescent mental health and social development. Depressive symptoms were measured using the affective problems subscale of the Youth Self Report Scale at comparable ages to those included in ALSPAC (Oldehinkel et al. 2015). Blood samples or buccal swabs were collected around age 16. DNA extraction and genotyping of SNPs were carried out at the Department of Genetics, University Medical Center Groningen, The Netherlands. Illumina HumanCytoSNP12v2 beadchip assay (Illumina, Inc; San Diago, CA, USA) was used to perform genome-wide genotyping. Impute v2(Howie, Donnelly, and Marchini 2009) was used to perform imputation with the 1000 Genomes reference panel (release March 2012) (Bastiaansen et al. 2015).

TEDS is a UK based longitudinal cohort of twin pairs focusing on cognitive and behavioural development. The replication analyses used self-reported SMFQ assessed when children were 12(Haworth, Davis, and Plomin 2013). Buccal swabs were collected and DNA extraction carried out by Affymetrix, Santa Clara, CA, USA. Genotyping was carried out using AffymetrixGeneChip 6.0 SNP genotyping arrays and raw image data were normalized and pre-processed at the Wellcome Trust Sanger Institute, Hinxton, UK. Genotypes for the arrays were called with CHIAMO. After quality control measures were applied, imputation was carried out using Impute v2(Howie, Donnelly, and Marchini 2009) using 1000 Genomes SNP data (Trzaskowski et al. 2013).

Power calculations for subgroup analyses

When performing our exploratory analyses stratified on sex and pubertal status, power to detect effects was diminished. Therefore, although these findings suggest some interesting hypotheses for future follow up, no firm conclusions can currently be made. Sample sizes for analyses stratified on sex ranged from 1414 at age 18, to 2774 at age 11. These analyses would have around 12% and 32% power respectively, to detect a true heritability of 17%. When also stratifying on puberty, sample drop to between 73 and 1673. Assuming a heritability of 17%, these analyses would have between 5% and 15% power to detect an effect.

**References**

Bastiaansen, Jojanneke A, Tarrant D R Cummins, Harriëtte Riese, Arie M van Roon, Ilja M Nolte, Albertine J Oldehinkel, and Mark A Bellgrove. 2015. “A Population Based Study of the Genetic Association between Catecholamine Gene Variants and Spontaneous Low-Frequency Fluctuations in Reaction Time.” *PloS One* 10 (5). Public Library of Science: e0126461. doi:10.1371/journal.pone.0126461.

Haworth, Claire M A, Oliver S P Davis, and Robert Plomin. 2013. “Twins Early Development Study (TEDS): A Genetically Sensitive Investigation of Cognitive and Behavioral Development from Childhood to Young Adulthood.” *Twin Research and Human Genetics : The Official Journal of the International Society for Twin Studies* 16 (1). Cambridge University Press: 117–25. doi:10.1017/thg.2012.91.

Howie, Bryan N, Peter Donnelly, and Jonathan Marchini. 2009. “A Flexible and Accurate Genotype Imputation Method for the next Generation of Genome-Wide Association Studies.” *PLoS Genetics* 5 (6). Public Library of Science: e1000529. doi:10.1371/journal.pgen.1000529.

Lee, S Hong, Jian Yang, Guo-Bo Chen, Stephan Ripke, Eli A Stahl, Christina M Hultman, Pamela Sklar, et al. 2013. “Estimation of SNP Heritability from Dense Genotype Data.” *American Journal of Human Genetics* 93 (6): 1151–55. doi:10.1016/j.ajhg.2013.10.015.

McMahon, George, Susan M Ring, George Davey-Smith, and Nicholas J Timpson. 2015. “Genome-Wide Association Study Identifies SNPs in the MHC Class II Loci That Are Associated with Self-Reported History of Whooping Cough.” *Human Molecular Genetics* 24 (20): 5930–39. doi:10.1093/hmg/ddv293.

Oldehinkel, Albertine J, Judith Gm Rosmalen, Jan K Buitelaar, Hans W Hoek, Johan Ormel, Dennis Raven, Sijmen A Reijneveld, et al. 2015. “Cohort Profile Update: The TRacking Adolescents’ Individual Lives Survey (TRAILS).” *International Journal of Epidemiology* 44 (1): 76–76n. doi:10.1093/ije/dyu225.

Speed, Doug, Gibran Hemani, Michael R Johnson, and David J Balding. 2012. “Improved Heritability Estimation from Genome-Wide SNPs.” *American Journal of Human Genetics* 91 (6): 1011–21. doi:10.1016/j.ajhg.2012.10.010.

Speed, Doug, Gibran Hemani, Michael R Johnson, and David J Balding. 2013. “Response to Lee et Al.: SNP-Based Heritability Analysis with Dense Data.” *American Journal of Human Genetics* 93 (6): 1155–57. doi:10.1016/j.ajhg.2013.10.016.

Trzaskowski, Maciej, Thalia C Eley, Oliver S P Davis, Sophia J Doherty, Ken B Hanscombe, Emma L Meaburn, Claire M A Haworth, Thomas Price, and Robert Plomin. 2013. “First Genome-Wide Association Study on Anxiety-Related Behaviours in Childhood.” *PloS One* 8 (4). Public Library of Science: e58676. doi:10.1371/journal.pone.0058676.

Visscher, P M, G Hemani, A A Vinkhuyzen, G B Chen, S H Lee, N R Wray, M E Goddard, and J Yang. 2014. “Statistical Power to Detect Genetic (Co)variance of Complex Traits Using SNP Data in Unrelated Samples.” *PLoS Genet* 10 (4): e1004269. doi:10.1371/journal.pgen.1004269.

Yang, Jian, Beben Benyamin, Brian P McEvoy, Scott Gordon, Anjali K Henders, Dale R Nyholt, Pamela A Madden, et al. 2010. “Common SNPs Explain a Large Proportion of the Heritability for Human Height.” *Nat Genet* 42 (7). Nature Publishing Group, a division of Macmillan Publishers Limited. All Rights Reserved.: 565–69. doi:http://www.nature.com/ng/journal/v42/n7/suppinfo/ng.608_S1.html.

**Additional Tables**

Table S1. Estimates adjusted for age at menarche

|  | Age 11 | | | Age 13 | | |
| --- | --- | --- | --- | --- | --- | --- |
|  | N | h^2^ (se) | P-value | N | h^2^ (se) | P-value |
| All females | 2515 | 0.12 (0.13) | 0.356 | 2459 | 0.01 (0.13) | 0.936 |
| Pre-puberty | 1133 | 0.45 (0.28) | 0.110 | 66 | <0.001 (5.24) | 1.000 |
| Post-puberty | 837 | 0.23 (0.39) | 0.561 | 1631 | 0.18 (0.20) | 0.370 |

Table S2. Heritability estimates of quantile normalized SMFQ scores in the overall ALSPAC sample adjusted for age and gender

| Age 11 | | | Age 13 | | | Age 18 | | |  |
| --- | --- | --- | --- | --- | --- | --- | --- | --- | --- |
| N | h^2^ (se) | P-value | N | h^2^(se) | P-value | N | h^2^(se) | P-value | |
| 5479 | 0.10 (0.06) | 0.087 | 5055 | 0.15 (0.06) | 0.017 | 3288 | 0.05 (0.10) | 0.631 | |

**Additional Figures**

Figure S1. Heritability estimates of depressive symptoms measured across childhood and adolescence. Estimates for both self-reported and parent-reported measures are displayed for ALSPAC and NTR.


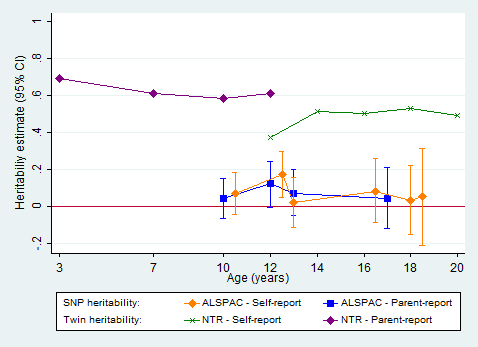


Figure S2. Distribution of self-reported SMFQ depression symptoms measured at 11, 13 and 18 years in ALSPAC


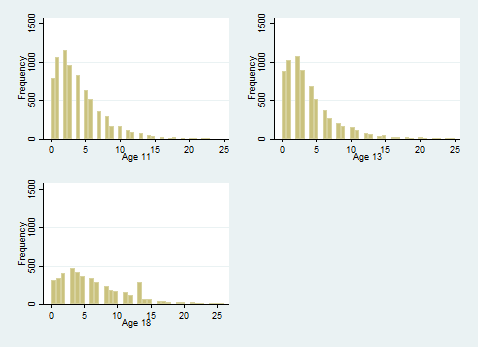


Figure S3. QQ plot for GWAS of self-reported SMFQ scores at age 13 in ALSPAC
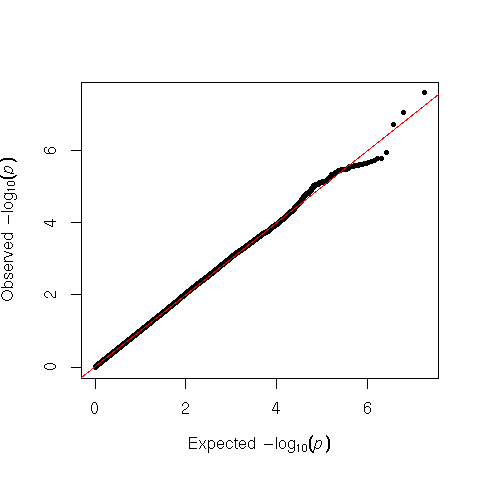


Figure S4. Forest plot showing beta coefficients of association between rs138191010 (risk allele: T) in ALSPAC GWAS at age 13 and depressive symptoms for each cohort
